# Supplementary material for: Towards Automation in IVF: Pre-Clinical Validation of a Deep Learning-Based Embryo Grading System during PGT-A Cycles
Source: J Clin Med. 2023 Feb 23;12(5):1806. doi: 10.3390/jcm12051806 (PMC10002983; doi:10.3390/jcm12051806)
Supplement: Supplementary file 1 [file jcm-12-01806-s001.zip › jcm-2147517-supplementary.pdf]

Supplementary Figure S1. iDAScore v1.0 decreases with increasing time of biopsy in hours post insemination (hpi). (A) Dispersion plot showing iDAScore v1.0 decrease according to the time of biopsy with 95% CI. (B) Dispersion showing iDAScore v1.0 decrease according to the time of biopsy in the four sub-group according to overall blastocyst quality (AA [excellent quality blastocysts] in green; AB or BA [good quality blastocysts] in blue; AC, CA, or BB [average quality blastocysts] in gold; BC, BC, or CC [poor quality blastocysts] in grey).

Blastocyst quality was defined according to Gardner's score adapted by Capalbo et al [13].

The dotted black lines identify the threshold for day 5 (≤120 hpi), day 6 (121-144 hpi) and day 7 (>144 hpi).

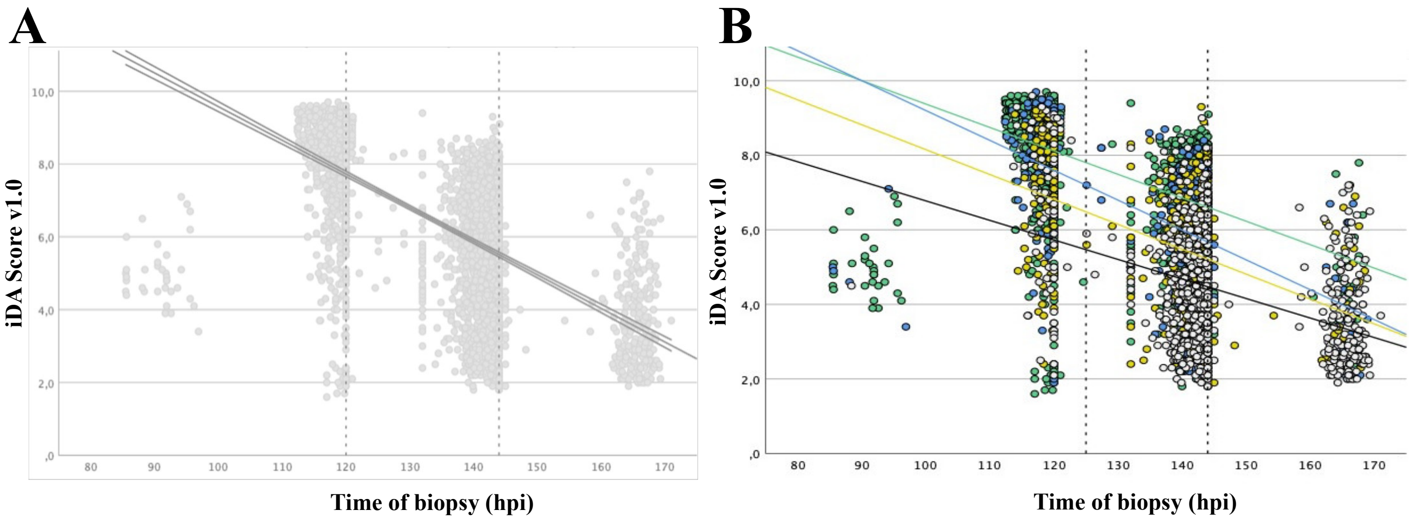

Linear regression:

Unstandardized coefficient B: -0.092, 95%CI from -0.096 to -0.089, p<0.01

Linear regressions:

- AA: Unstandardized coefficient B: -0.063, 95%CI from -0.068 to -0.057, p<0.01
- AB, BA: Unstandardized coefficient B: -0.080, 95%CI from -0.090 to -0.071, p<0.01
- BB, AC, CA: Unstandardized coefficient B: -0.067, 95%CI from -0.078 to -0.055, p<0.01
- CC, BC, CB: Unstandardized coefficient B: -0.052, 95%CI from -0.061 to -0.044, p<0.01

Supplementary Figure S2. iDAScore v1.0 decreases with increasing maternal age (with 95% CI)**.**

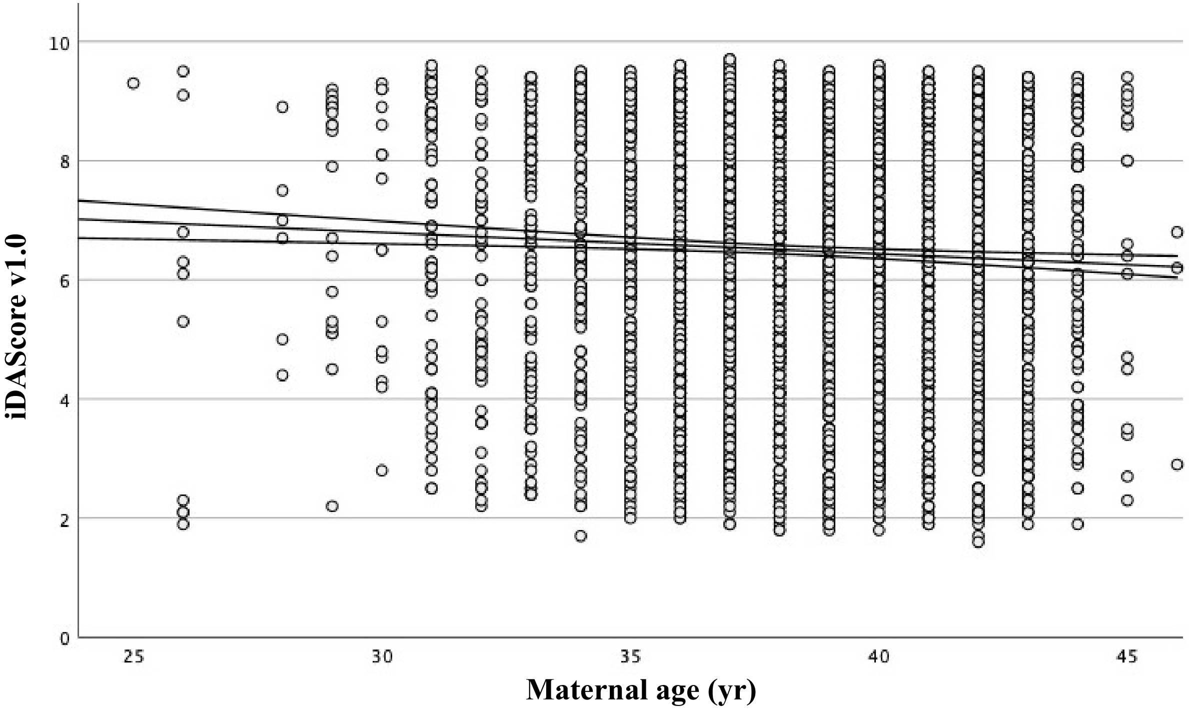

Linear regression

Unstandardized coefficient B: -0.036, 95%CI from -0.057 to -0.015, p<0.01

Supplementary Figure S3. (A) iDAScore v1.0 decreases according to the time of biopsy (in hours post insemination, hpi) **in the three clusters of blastocyst's chromosomal constitution** (euploid, green; single aneuploid, light red; complex aneuploid, dark red)**. In the sub-group analyses, (B) iDAScore v1.0 were similar between euploid and aneuploid blastocysts across the 4 clusters of overall blastocyst quality as defined by the embryologists** (AA [excellent quality blastocysts]; AB or BA [good quality blastocysts]; AC, CA, or BB [average quality blastocysts]; BC, BC, or CC [poor quality blastocysts])**, while (C) it was still significantly different among blastocysts clustered as day 5** (≤120 hpi) **or day 6** (121-144 hpi) (for day 7 blastocysts, >144 hpi, the p-value was >0.05).

Blastocyst quality was defined according to Gardner's score adapted by Capalbo et al [13].

The dotted black lines identify the threshold for day 5 (≤120 hpi), day 6 (121-144 hpi) and day 7 (>144 hpi).

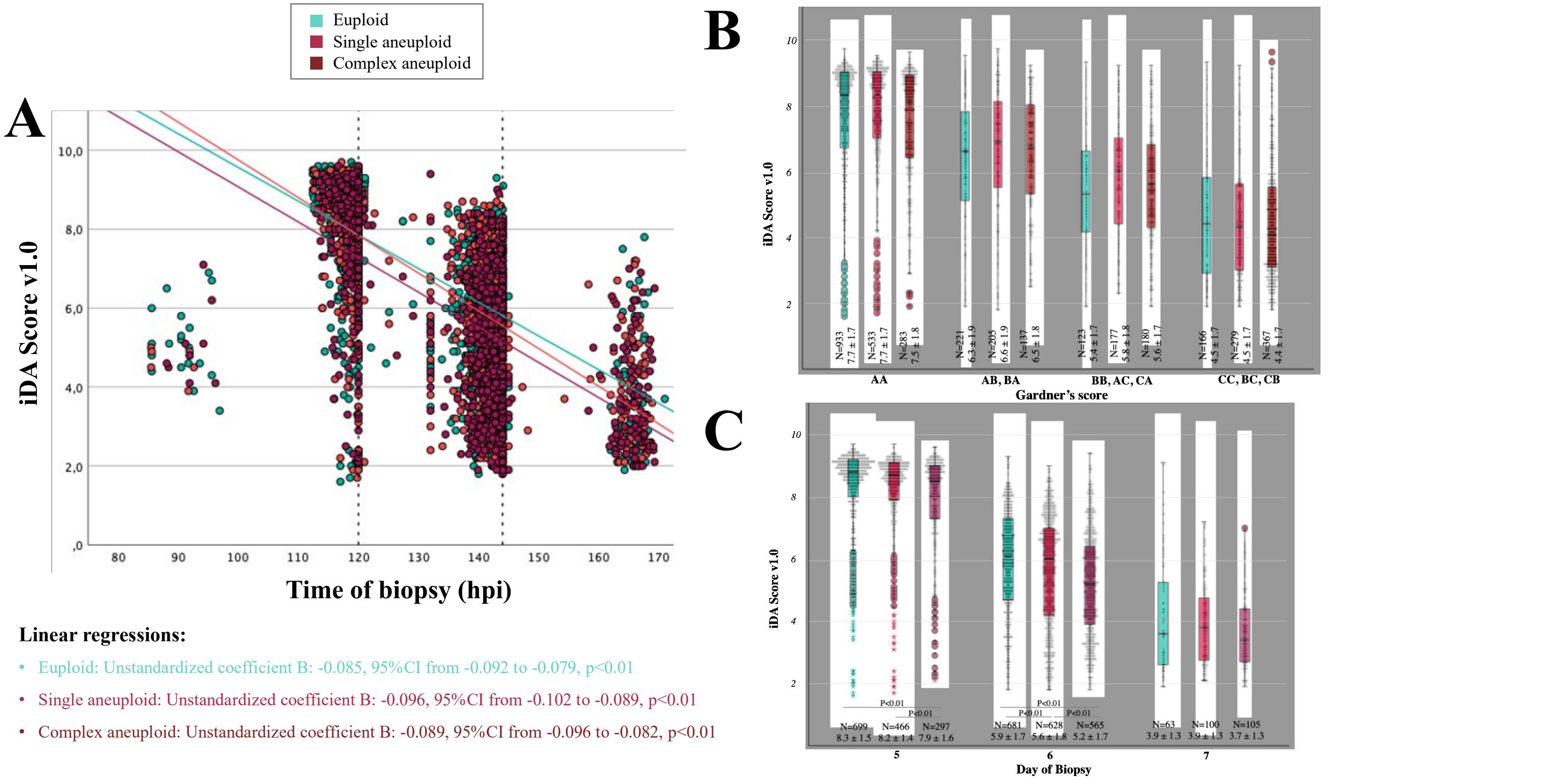

Supplementary Figure S4. (A) iDAScore v1.0 shows a sharper decrease according to the time of biopsy (in hours post insemination, hpi) **among euploid blastocysts that did not result in a live birth** (LB, orange) **than among the ones that did result in a LB** (light green)**. In the sub-group analyses, (B) iDAScore v1.0 were significantly different in the "no LB" and "LB" groups further clustered according to the overall blastocyst quality as defined by the embryologists** (AA [excellent quality blastocysts]; AB or BA [good quality blastocysts]; AC, CA or BB [average quality blastocysts]; for BC, BC or CC [poor quality blastocysts] p-value was >0.05). **Similarly, (C) iDAScores v1.0 were significantly different in the "no LB" and "LB" groups further clustered according to the day of biopsy** (day 5, ≤120 hpi; day 6, 121-144 hpi; for day 7 blastocysts, >144 hpi, the p-value was >0.05).

Blastocyst quality was defined according to Gardner's score adapted by Capalbo et al [13].

The dotted black lines identify the threshold for day 5 (≤120 hpi), day 6 (121-144 hpi) and day 7 (>144 hpi).

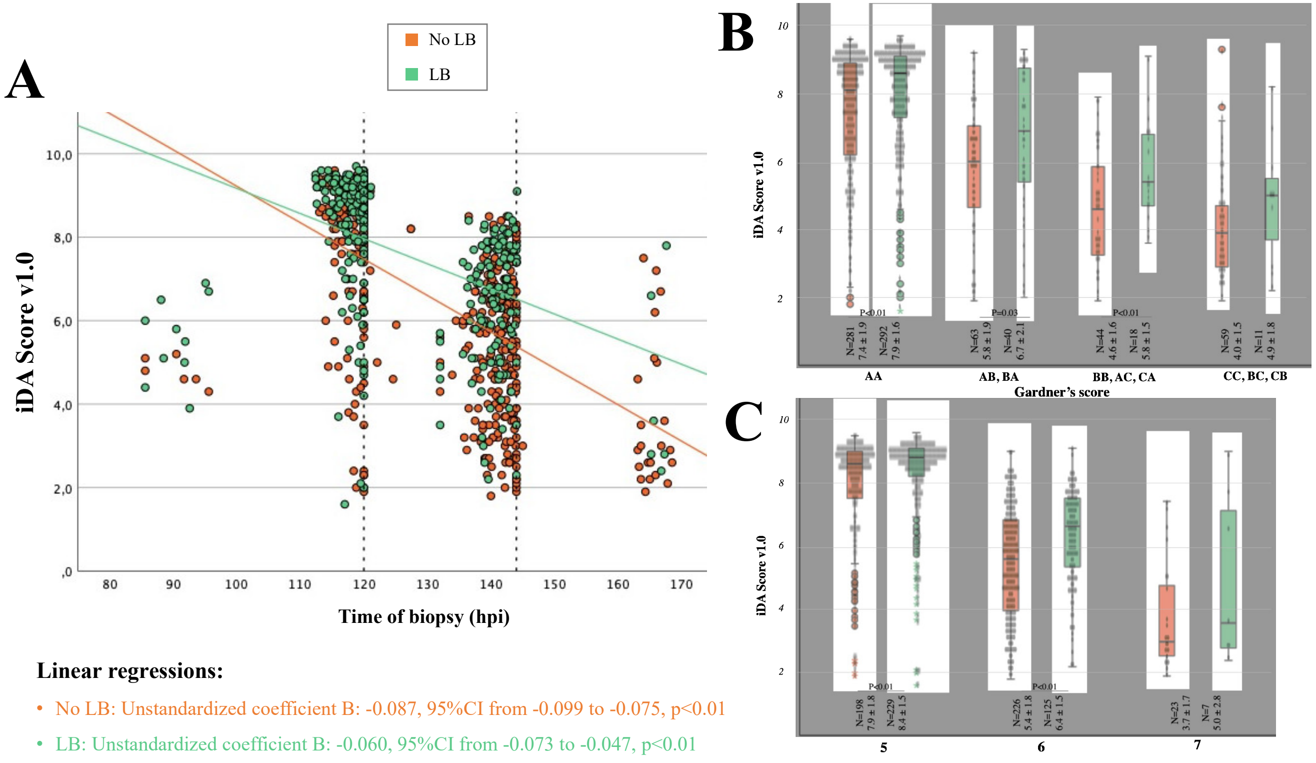

Supplementary Figure S5. Simulation of iDAScore v1.0 clinical utility had we use it in the context of IVF cycles with preimplantation genetic testing for aneuploidies (PGT-A). In 52% of the cases where at least 2 euploid blastocysts were identified within each cohort, the embryologists and the software would have been equally effective in prioritizing the euploid competent blastocyst for transfer (yellow portion), while in 15% (green portion) and 3% (red portion) of the cases iDAScore v1.0 would have involved an earlier and a later live birth, respectively. However, in 29% of the cases we could not assess the putative influence of iDAScore v1.0 (being either equally effective or involving a later live birth) since a better-ranked euploid blastocyst has not yet been transferred, while a live birth was achieved with another lower-ranked euploid blastocyst from the same cohort (grey portion).

The raw data used for these calculations are shown in Supplementary Table 2.

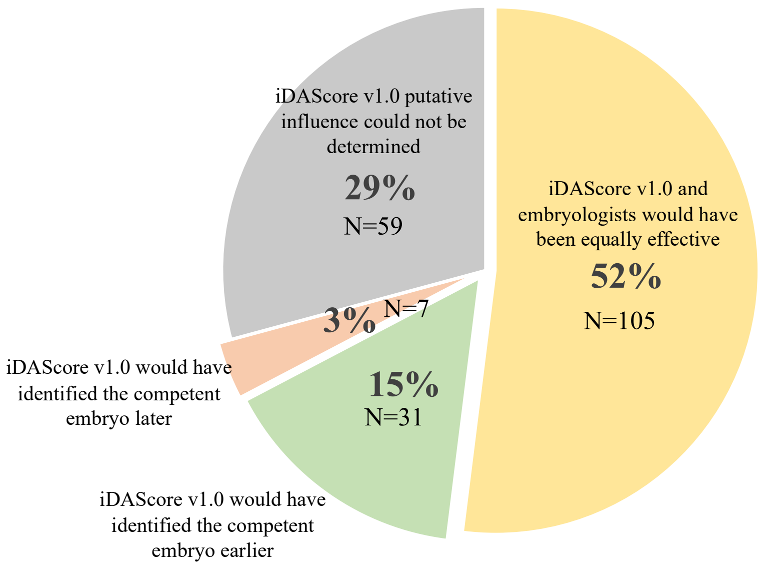

Supplementary Table S1. Patients and cycles characteristics

BMI, body mass index; OAT, oligoasthenoteratozoospermic; COC, cumulus oocyte complex

**Patients included, N**	**1232**
**Maternal age, mean ± SD (yr)**	38.7 ± 3.4
**BMI, mean ± SD**	21.9 ± 3.2
**Previous conceptions, N, %**	
**No**	872, 70.8%
**Yes**	360, 29.2%
**Duration of infertility, mean ± SD (yr)**	3.5 ± 2.3
**Main cause of infertility, N, %:**	
**Idiopathic**	813, 66%
**Endocrine-ovulatory**	34, 2.8%
**Endometriosis**	90, 7.3%
**Tubal**	100, 8.1%
**Male factor**	195, 15.8%
**Semen characteristics, N, %**	
**Normozoospermic**	678, 55%
**1-2 defects**	331, 26.9%
**OAT**	213, 17.3%
**Surgical**	10, 0.8%
**COCs retrieved, mean ± SD**	10.7 ± 6.4
**Biopsied blastocysts, mean ± SD**	2.9 ± 1.8
**Euploid blastocysts, mean ± SD**	1.2 ± 1.3

Supplementary Table S2. Raw data for the concordance between the operators and the iDA Score v1.0 in ranking euploid blastocysts for transfer. We included all cycles between April 2013 and December 2021 where at least 2 euploid embryos were obtained, at least one live birth was achieved, and the ranking outlined by the iDA Score v1.0 could be assessed as beneficial or not. The euploid blastocysts are listed according to the rank outlined by the iDA Score v1.0 (the green color pinpoints embryos that resulted in a live birth, the orange color pinpoints embryos that did not result in a live birth, the white color pinpoint embryos that had not been transferred yet before manuscript drafting). Every column near by the iDA Score v1.0 reports the consecutive number of transfers according to the choice made by the operators (who were blinded to the iDA Score v1.0). The last column identifies cases where iDA Score v1.0 and operators would have been equally effective, cases where the iDA Score v1.0 would have involved an earlier live birth, cases where the iDA Score v1.0 would have caused a later live birth, and cases where the putative influence of the iDA Score v1.0 could not be assessed because euploid blastocysts ranked higher have not been transferred yet, although a live birth was already achieved with euploid blastocysts ranked lower (?).

**Couple**	1st	ET N	2nd	ET N	3rd	ET N	4th	ET N	5th	ET N	6th	ET N	7th	ET N	8th	ET N	9th	ET N	10th	ET N	11th	ET N	iDA Score v1.0 vs operator
**1**	6.0	1	5.1	2	4.8	3	4.4	4															Equally effective
**2**	9.2	1	8.7	2	6.7	3																	Equally effective
**3**	3.5	1	3.0	2																			Equally effective
**4**	8.6	1	6.2	2	4.9																		Equally effective
**5**	8.9	1	8.9	2																			Equally effective
**6**	3.6	1	2.8	2																			Equally effective
**7**	9.0	1	8.7	2																			Equally effective
**8**	9.1	1	9.1	2	8.9		7.6		4.1														Equally effective
**9**	9.4	1	9.3	2																			Equally effective
**10**	9.4	1	6.5	2																			Equally effective
**11**	6.5	1	5.9	2																			Equally effective
**12**	9.3	1	6.2	2	5.9																		Equally effective
**13**	9.0	1	8.9	2	6.8																		Equally effective
**14**	8.8	1	6.8	2																			Equally effective
**15**	9.4	1	8.9	2	5.9																		Equally effective
**16**	5.1	1	3.5	2																			Equally effective
**17**	9.7	1	9.2	2																			Equally effective
**18**	7.3	1	4.9	2																			Equally effective
**19**	8.9	1	3.6	2																			Equally effective
**20**	6.5	1	4.2	2																			Equally effective

**21**	7.4	1	4.1	2																			Equally effective
**22**	5.5	1	3.0	2																			Equally effective
**23**	8.8	1	8.0	2	6.2																		Equally effective
**24**	9.5	1	9.4	2	9.0		8.9		4.8														Equally effective
**25**	9.1	1	4.3	2																			Equally effective
**26**	9.6	1	8.9	2																			Equally effective
**27**	5.9	1	5.3	2																			Equally effective
**28**	7.5	1	4.7	2	3.7																		Equally effective
**29**	8.9	1	8.8		8.5		3.9	2															Equally effective
**30**	9.3	1	4.5		2.4																		Equally effective
**31**	5.1	1	4.9																				Equally effective
**32**	9.1	1	7.1																				Equally effective
**33**	9.1	1	5.1																				Equally effective
**34**	7.3	1	7.0		4.9																		Equally effective
**35**	8.2	1	7.2		7.1		5.9																Equally effective
**36**	9.4	1	5.8																				Equally effective
**37**	6.3	1	5.4																				Equally effective
**38**	9.4	1	5.6		4.7																		Equally effective
**39**	8.6	1	5.0																				Equally effective
**40**	6.9	1	5.0																				Equally effective
**41**	7.6	1	4.9																				Equally effective
**42**	8.9	1	8.3																				Equally effective
**43**	9.4	1	9.0		7.5																		Equally effective
**44**	6.8	1	4.0																				Equally effective
**45**	6.3	1	5.2		4.3																		Equally effective
**46**	8.0	1	6.3		4.5																		Equally effective
**47**	9.2	1	5.6																				Equally effective
**48**	9.4	1	9.2		9.2		4.0																Equally effective
**49**	8.7	1	8.5																				Equally effective
**50**	7.0	1	6.4		6.1																		Equally effective
**51**	9.4	1	8.9		6.0																		Equally effective

**52**	9.2	1	8.9																				Equally effective
**53**	6.5	1	5.6		2.8																		Equally effective
**54**	7.4	1	7.0																				Equally effective
**55**	9.0	1	8.9		6.9																		Equally effective
**56**	8.9	1	4.3																				Equally effective
**57**	7.9	1	5.8																				Equally effective
**58**	5.5	1	5.0		4.5																		Equally effective
**59**	9.6	1	8.4		3.1																		Equally effective
**60**	6.7	1	4.8																				Equally effective
**61**	9.2	1	8.7																				Equally effective
**62**	9.4	1	7.3																				Equally effective
**63**	8.1	1	6.3																				Equally effective
**64**	9.0	1	5.5																				Equally effective
**65**	9.0	1	7.4		6.6		6.0																Equally effective
**66**	9.1	1	6.7																				Equally effective
**67**	9.0	1	6.8		4.5																		Equally effective
**68**	7.9	1	7.8		6.1																		Equally effective
**69**	7.9	1	7.2																				Equally effective
**70**	9.2	1	8.9		8.5		7.0		5.1		3.6												Equally effective
**71**	8.0	1	7.0		6.6																		Equally effective
**72**	6.5	1	4.7																				Equally effective
**73**	3.9	1	2.1																				Equally effective
**74**	9.4	1	8.8		8.2		6.5																Equally effective
**75**	8.6	1	4.6																				Equally effective
**76**	8.8	1	8.2		7.3																		Equally effective
**77**	9.0	1	7.1																				Equally effective
**78**	6.5	1	5.3																				Equally effective
**79**	9	1	7.6																				Equally effective
**80**	9.3	1	9.2		9.2		8.9		7.4		6.2												Equally effective
**81**	9.4	1	9.4																				Equally effective
**82**	9.2	1	9.0																				Equally effective

**83**	9.3	1	8.1		8.1																		Equally effective
**84**	8.5	1	7.9																				Equally effective
**85**	8.1	1	6.9																				Equally effective
**86**	8.9	1	7.6																				Equally effective
**87**	8.8	1	8.6		6.2																		Equally effective
**88**	9.4	1	8.4		6.4																		Equally effective
**89**	9.2	1	4.4		4.2																		Equally effective
**90**	9	1	6.1																				Equally effective
**91**	7.7	1	6.5																				Equally effective
**92**	8.6	1	8.1		7.5																		Equally effective
**93**	9.1	1	8.4		8.1		8.0		7.1														Equally effective
**94**	9.3	1	9.1																				Equally effective
**95**	8.2	1	7.1																				Equally effective
**96**	8.5	1	5.8																				Equally effective
**97**	8.4	2	7.0	1																			Equally effective
**98**	9.3	2	5.0	1																			Equally effective
**99**	9.0	2	8.6	1	6.1		5.4		3.9		3.8												Equally effective
**100**	9.4	2	9.2	1	8.2		6.4		5.2														Equally effective
**101**	9.4	2	7.6	1																			Equally effective
**102**	9.5	2	9.1		8.5		7.9	1	7.6		3.4												Equally effective
**103**	9.2	3	8.8	2	7.2	1																	Equally effective
**104**	9.0	3	9.0	2	8.3		7.4	1	6.9														Equally effective
**105**	9.3	2	8.6	1	7.8	3																	Equally effective
**106**	9.1	2	7.7	1	6.9	3	5.5	4															Later live birth
**107**	8.8	2	8.7	1	6.2	3																	Later live birth
**108**	5.3	2	4.1	1																			Later live birth
**109**	6.4	2	5.7	1																			Later live birth
**110**	8.9	2	6.5	1																			Later live birth
**111**	7.7	2	6.6	1	4.4																		Later live birth
**112**	9.4	2	7.9	1																			Later live birth
**113**	9.3	3	9.3	2	6.6	1																	Earlier live birth

**114**	7.7	4	5.8	3	3.2	1	2.5	2															Earlier live birth
**115**	9.3	1	9.1	3	7.0	2																	Earlier live birth
**116**	9.3	1	7.8	3	6.7	2																	Earlier live birth
**117**	8.5	2	8.2	1	6.8		6.6	4	6.4		3.9	3	2.8										Earlier live birth
**118**	8.9	2	7.0	1																			Earlier live birth
**119**	6.7	2	6.5	1																			Earlier live birth
**120**	9.1	2	7.9	1	7.4		6.2																Earlier live birth
**121**	9.2	2	8.6	1	6.7		6.3																Earlier live birth
**122**	6.9	2	5.8	1																			Earlier live birth
**123**	6.8	2	6.0	1																			Earlier live birth
**124**	9.3	2	7.9	1																			Earlier live birth
**125**	9.5	2	8.2	1																			Earlier live birth
**126**	9.5	2	9.0	1	9.0		8.6																Earlier live birth
**127**	5.0	2	4.6	1																			Earlier live birth
**128**	5.0	2	1.8	1																			Earlier live birth
**129**	8.3	2	7.6	1	5.8																		Earlier live birth
**130**	7.9	2	5.0	1																			Earlier live birth
**131**	9.1	2	3.1	1																			Earlier live birth
**132**	9.0	2	7.5	3	3.4	1	2.7	4															Earlier live birth
**133**	9.1	2	9.0	3	8.8	1	7.3		3.2														Earlier live birth
**134**	7.6	2	7.3	3	7.0	1	5.3		4.3														Earlier live birth
**135**	9.3	2	9.3		9.1	1	8.9		8.0		6.5												Earlier live birth
**136**	5.8	2	5.3		5.2	1	4.5																Earlier live birth
**137**	9.3	2	9.2		8.6	1	7.7																Earlier live birth
**138**	6.6	2	4.8		2.0	1																	Earlier live birth
**139**	8.4	3	7.0	1	4.6	2																	Earlier live birth
**140**	8.8	3	8.0	1	7.9	2	3.5																Earlier live birth
**141**	7.4	3	5.9	1	5.2	2																	Earlier live birth
**142**	5.8	3	5.6	1	5.4		5.1		4.7	2	4.7												Earlier live birth
**143**	9.3	3	8.7	1	8.4		7.7		5.6	2													Earlier live birth
**144**	9.4	1	8.9	2	8.9		6.0	3															?

**145**	8.8	1	8.8		8.7	2																	?
**146**	8.9	1	7.6		3.6	2																	?
**147**	9.1	1	5.8		5.3		4.8	2															?
**148**	8.5	1	6.3		6.3		6.3		6.2	2	5.7		4.9										?
**149**	5.4		2.3	1	2.3	2	2.0	3															?
**150**	9.2		9.2	1	7.2	2	6.2	3															?
**151**	8.1		6.7	1	5.8	2	4.0																?
**152**	6.2		5.9	1	3.2	2																	?
**153**	9.3		9.2	1	9.2	2	8.9		7.9														?
**154**	9.4		9.2	1	9.2	2	8.5																?
**155**	9.2		9.0	1	8.7		6.3	2															?
**156**	5.3		4.3	1	4.0																		?
**157**	7.8		4.2	1																			?
**158**	6.3		6.1	1																			?
**159**	3.1		2.8	1																			?
**160**	7.3		4.7	1																			?
**161**	8.8		3.7	1																			?
**162**	9.2		8.3	1	2.7																		?
**163**	6.9		6.8	1	3.5		2.4																?
**164**	9.1		9.0	1	4.2																		?
**165**	9.3		9.2	1	4.0																		?
**166**	7.5		6.8	1	5.8																		?
**167**	7.5		4.7	1																			?
**168**	7.8		6.3	1																			?
**169**	9.3		9.0	1	8.6		4.6		4.4		4.2												?
**170**	6.7		5.0	1																			?
**171**	7.6		7.1	1	3.9																		?
**172**	3.8		3.4	1																			?
**173**	8.3		8.0	1	5.6																		?
**174**	9.3		8.6	1	6.1																		?
**175**	9.1		8.2	1	6.0																		?

**176**	9.5		8.7	1																			?
**177**	9.0		8.9	1	8.8		8.6		7.9														?
**178**	9.2		7.6	1																			?
**179**	2.0		1.6	1																			?
**180**	9.0		8.8	1	6.7																		?
**181**	8.9		8.5	1	6.1																		?
**182**	9.1		9.1	1	8.8		8.5		8.4		7.1		6.8										?
**183**	9.4		9.3	1																			?
**184**	9.3		8.8	1																			?
**185**	8.3		8.3	1	8.1																		?
**186**	8.8		8.6	1																			?
**187**	9.5		9.2	1	9.0		8.4																?
**188**	5.7		5.0	1	2.4																		?
**189**	9.2		9.1	1	8.8		8.7		8.7		8.6		8.5										?
**190**	7.9		7.5	1																			?
**191**	8.4		7.1	1	4.4																		?
**192**	9.6		9.4	1	9.1																		?
**193**	5.1		4.6	2	4.6	1	4.3																?
**194**	8.0		7.6		5.9	1																	?
**195**	8.3		6.7		6.5	1																	?
**196**	6.9		6.8		6.0	1																	?
**197**	9.0		9.0		8.9	1	3.5																?
**198**	8.7		8.1		7.5	1	7.2		6.4														?
**199**	7.7		5.8		5.7	2	3.9	1															?
**200**	7.8		7.4		7.3	1	7.2																?
**201**	9.4		9.3		9.2		9.1	1	9.1		8.9		8.8		8.2	2	7.6		6.3		3.4		?
**202**	8.9		8.4		5.9		3.5	1															?
